# Bta-miR-146a Inhibits Proliferation and Promotes Apoptosis of Bovine Immature Sertoli Cells by Targeting SMAD4 via the TGF-β/MAPK Signaling Pathway

**DOI:** 10.3390/ijms27177554

**Published:** 2026-08-24

**Authors:** Qiwen Lu, Quanheng Guo, Yanlong Zhou, Qiuyan Tao, Ruiwen Chen, Qianchao Xu, Zhihui Zhao, Ping Jiang

**Affiliations:** College of Coastal Agricultural Sciences, Guangdong Ocean University, Zhanjiang 524088, China; 3366lao@gmail.com (Q.L.); gheng2022@163.com (Q.G.); 16638772921@stu.gdou.edu.cn (Y.Z.); 15651760800@stu.gdou.edu.cn (Q.T.); w138222373092@163.com (R.C.); 166603730974@stu.gdou.edu.cn (Q.X.); zhzhao@gdou.edu.cn (Z.Z.)

**Keywords:** Chinese Holstein cattle, Bta-miR-146a, immature Sertoli cells, SMAD4, transforming growth factor-β/mitogen-activated protein kinase (TGFβ/MAPK), proliferation, apoptosis

## Abstract

Sertoli cells (SCs) are essential for spermatogenesis and provide structural and nutritional support to germ cells in the Chinese Holstein cattle testis. Although microRNAs (miRNAs) are known to regulate SC function, the specific role of Bta-miR-146a in bovine SCs is unclear. This study investigated the mechanisms by which Bta-miR-146a regulates bovine immature SCs. Using molecular cloning, we constructed Bta-miR-146a overexpression and interference vectors and transfected them into SCs via lipofection. Quantitative real-time PCR (RT-qPCR), 5-ethynyl-2′-deoxyuridine (EdU) proliferation assays, Cell Counting Kit-8 (CCK-8) viability assays, and flow cytometry revealed that Bta-miR-146a overexpression inhibited SC proliferation and promoted apoptosis, whereas Bta-miR-146a inhibition increased proliferation and suppressed apoptosis. Dual-luciferase reporter assays confirmed that SMAD4 is a direct target of Bta-miR-146a; SMAD4 interference reduced SC proliferation and increased apoptosis, whereas overexpression had the opposite effect. Furthermore, activity of this gene modulates the TGFβ/MAPK signaling pathway; SMAD4 interference reduces the expression of TGFβ, TGF-βRII, DAXX, MAP3K5, P38, and MAX. These findings indicate that the Bta-miR-146a/SMAD4/TGFβ/MAPK axis is a key regulator of SC proliferation and apoptosis, offering insights into the molecular mechanisms underlying bovine spermatogenesis and potential targets for improving reproductive performance.

## 1. Introduction

Sertoli cells (SCs) are essential somatic cells in the mammalian testis that play a critical role in spermatogenesis by providing structural support and nourishment to developing germ cells, creating a microenvironment conducive to their development [1]. In cattle, SCs are key to determining testis size and sperm production capacity, as their proliferative activity during the immature stage directly influences their final number in the mature testis [2]. This, in turn, impacts reproductive efficiency, a key factor in the cattle industry, where optimizing breeding performance is crucial for genetic improvement [3]. Despite advances in the understanding of bovine reproduction, the molecular mechanisms involved in the regulation of SC proliferation and apoptosis remain unclear, particularly in the context of epigenetic regulatory factors such as miRNAs.

MicroRNAs (miRNAs) are small noncoding RNAs that posttranscriptionally regulate gene expression by targeting the 3′ untranslated region (3′ UTR) of messenger RNAs (mRNAs), thereby modulating processes such as cell proliferation, apoptosis, and differentiation. In testicular SCs, miRNAs such as miR-133b and miR-495 have been shown to regulate proliferation and apoptosis by targeting specific genes, influencing spermatogenesis [4,5]. Our previous miRNA sequencing analysis of neonatal and adult Holstein bull testes revealed the differential levels of Bta-miR-146a, with higher levels in immature tissues, suggesting the potential role of this miRNA in modulating SC function during early testicular development.

miR-146a is a widely studied microRNA that plays a critical role in regulating cell proliferation, apoptosis, and inflammatory responses across diverse cell types by modulating key signaling pathways, particularly the transforming growth factor-β (TGF-β) pathway through direct targeting of SMAD4—a central mediator in TGF-β signal transduction [6,7,8]. For instance, miR-146a inhibits proliferation and promotes apoptosis in hepatic stellate cells [6], gastric cancer cells [9], and chondrocytes [7] by suppressing SMAD4 expression, thereby impairing TGF-β signaling. In bovine species, Bta-miR-146a shows differential expression in testicular tissues and is implicated in inflammatory regulation in mammary epithelial cells [10], but its precise function in SCs and specific molecular targets remain largely unexplored.

The SMAD4 gene, located in the 18q21.1 region of human chromosome 18 [11] with a syntenic counterpart on bovine chromosome 24, encodes a member of the SMAD family (SMAD family member 4). As the common-mediator SMAD(Co-SMAD) in the transforming growth factor-β (TGF-β) signaling pathway, it plays a critical role in signal transduction by forming complexes with receptor-regulated SMADs to regulate target gene expression [12]. Studies on SMAD4 have indicated that it is involved in increasing the expression of cell cycle inhibitors, such as p21 (cyclin-dependent kinase inhibitor 1A, CDKN1A) and p15 (cyclin-dependent kinase inhibitor 2B, CDKN2B), which suppress the activity of the Cyclin D-CDK4/6 complex, leading to cell cycle arrest at the G1 phase and thereby inhibiting proliferation [13]. However, the role of SMAD4 is not solely inhibitory; in certain contexts, it may indirectly promote proliferation through noncanonical pathways, such as synergistic interactions with the Wnt signaling pathway. For instance, SMAD4 mutations may increase β-catenin activity, promoting cell proliferation in colorectal cancer [14]. In testicular SCs, this dual functionality likely balances proliferation during development and maturation [15].

Apoptosis is another critical process regulated by SMAD4; it not only modulates apoptosis but also interacts with multiple apoptotic signaling pathways [16]. For example, in porcine follicular granulosa cells, SMAD4 directly binds to the Frizzled-4(FZD4) promoter region to increase the transcriptional activity of the latter, activating the Wnt signaling pathway to inhibit granulosa cell apoptosis [17]. Furthermore, SMAD4 influences apoptosis through crosstalk with other signaling pathways, such as the c-Jun N-terminal kinase (JNK) and mitogen-activated protein kinase (MAPK) pathways [18]. In pancreatic cancer cells, a deficiency in SMAD4 leads to hyperactivation of the JNK pathway, inhibiting apoptosis and promoting cell survival, whereas restoring SMAD4 expression reactivates pro-apoptotic signals [19]. These interactions highlight the context-dependent role of SMAD4 in balancing cell fate. As a central transcription factor in the TGF-β signaling pathway, SMAD4 acts as a co-SMAD, forming complexes with SMAD2/3 to regulate target gene transcription, thereby influencing cell proliferation, differentiation, and apoptosis [20]. TGF-β signaling operates through both canonical SMAD-dependent and noncanonical SMAD-independent pathways, the latter of which includes the p38 mitogen-activated protein kinase (p38-MAPK) pathway [21].

The p38-MAPK pathway, a key member of the MAPK family, is directed by a kinase cascade involving mitogen-activated protein kinase kinase kinase (MAP3K; e.g., apoptosis signal-regulating kinase 1 (ASK1) and transforming growth factor-β-activated kinase 1 (TAK1)), mitogen-activated protein kinase kinase (MAP2K; e.g., MKK3/6), and p38; phosphorylation of the latter at Thr180/Tyr182 drives its activation in response to stressors such as oxidative stress and inflammatory cytokines [22]. Crosstalk between the TGF-β and p38-MAPK pathways, mediated by SMAD4, has been implicated in various physiological and pathological processes, including the epithelial–mesenchymal transition (EMT), fibrosis, inflammation, and tumorigenesis [23,24,25]. SMAD4 may regulate p38-MAPK signaling by directly modulating genes linked to blood-testis barrier (BTB) integrity, with its knockout in mice leading to defective spermatogenesis and fewer SCs [26,27].

This study, based on small RNA sequencing (sRNA-Seq) of testicular tissues from neonatal and mature Chinese Holstein cattle, identified miRNAs associated with cell proliferation and apoptosis. We selected Bta-miR-146a as the primary research object and investigated its role in the proliferation and apoptosis of immature bovine testicular SCs by targeting the *SMAD4* gene, thereby influencing testicular development and spermatogenesis, and providing molecular insights to enhance cattle reproductive performance. Overexpression/knockdown vectors and dual-luciferase reporter assays will validate the Bta-miR-146a/SMAD4 axis and its downstream signaling pathways, offering a theoretical foundation for miRNA-based evaluation of breeding bull fertility and the development of high-fecundity livestock breeds.

## 2. Results

### 2.1. Transfection Efficiency of Bta-miR-146a Mimics and Inhibitors

**Figure 1:**
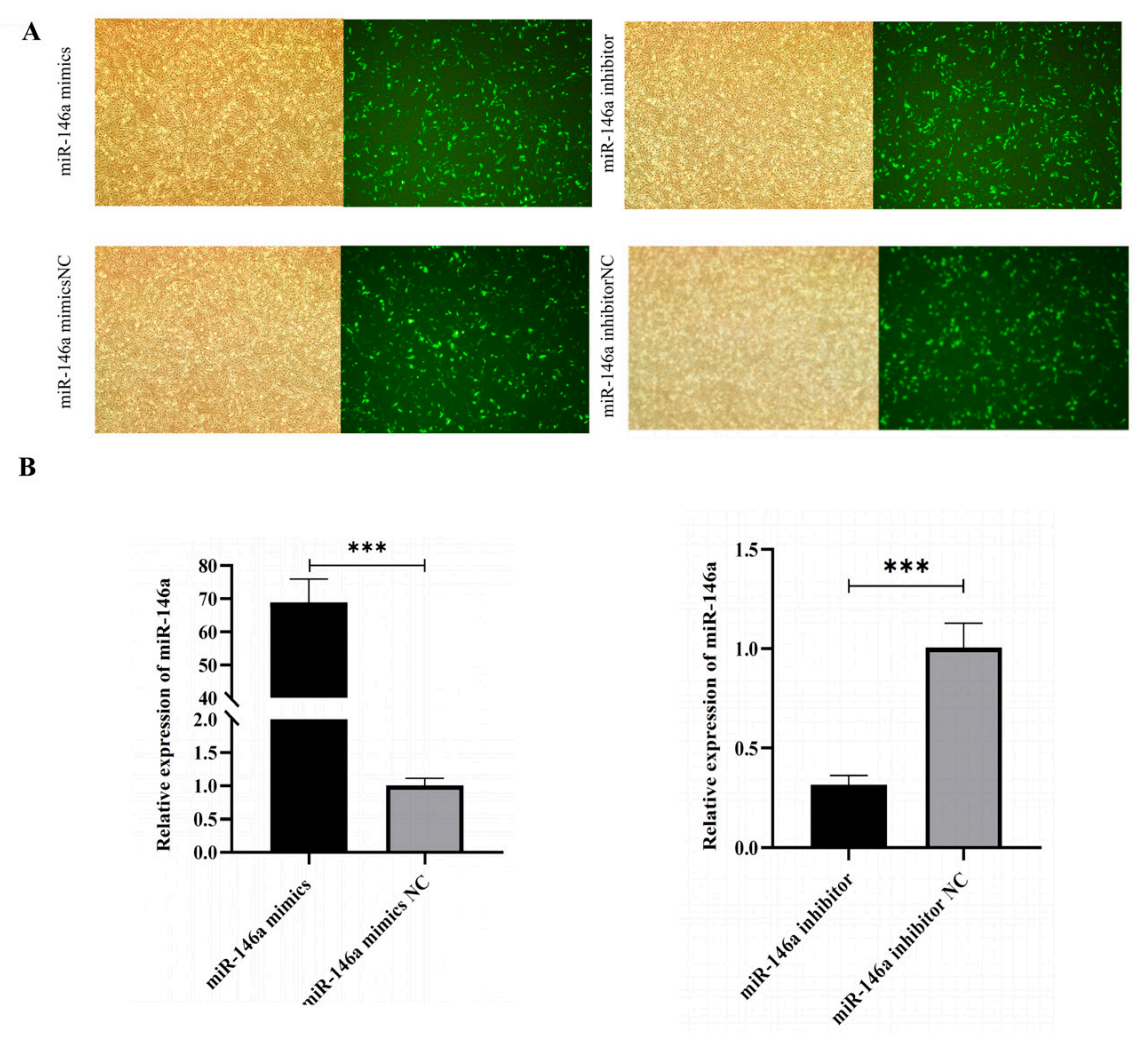
Transfection efficiency of Bta-miR-146a mimics and inhibitors in bovine immature SCs. (**A**) Representative fluorescence images showing transfection efficiency (green fluorescence indicates successful transfection with GFP-containing vectors). Images were captured at 4× magnification; scale bar = 100 μm, (**B**) RT-qPCR analysis of Bta-miR-146a levels in mimics, mimics NC, inhibitor, and inhibitor NC groups. Values are mean ± SEM (n = 3). ***, *p* < 0.001 versus respective NC.

To investigate the role of Bta-miR-146a in bovine SCs, Bta-miR-146a mimics and inhibitors and their respective negative controls (NCs) were transfected into bovine immature SCs. After 36 h of culture, the transfection efficiency was assessed using an inverted fluorescence microscope. As shown in Figure 1A, cells transfected with different plasmids exhibited comparable transfection efficiencies of approximately 70%. Total RNA was extracted and reverse transcribed into cDNA for RT-qPCR to measure Bta-miR-146a levels. The RT-qPCR results (Figure 1B) demonstrated that transfection with Bta-miR-146a mimics significantly increased Bta-miR-146a levels compared with that in the control group (*p* < 0.001). Conversely, transfection with the Bta-miR-146a inhibitor significantly reduced Bta-miR-146a levels compared with that in the inhibitor NC group (*p* < 0.01). These results confirm that the transfection protocol effectively modulated Bta-miR-146a levels in SCs, enabling further functional studies.

### 2.2. Bta-miR-146a Inhibits the Proliferation of Immature Bovine SCs

**Figure 2:**
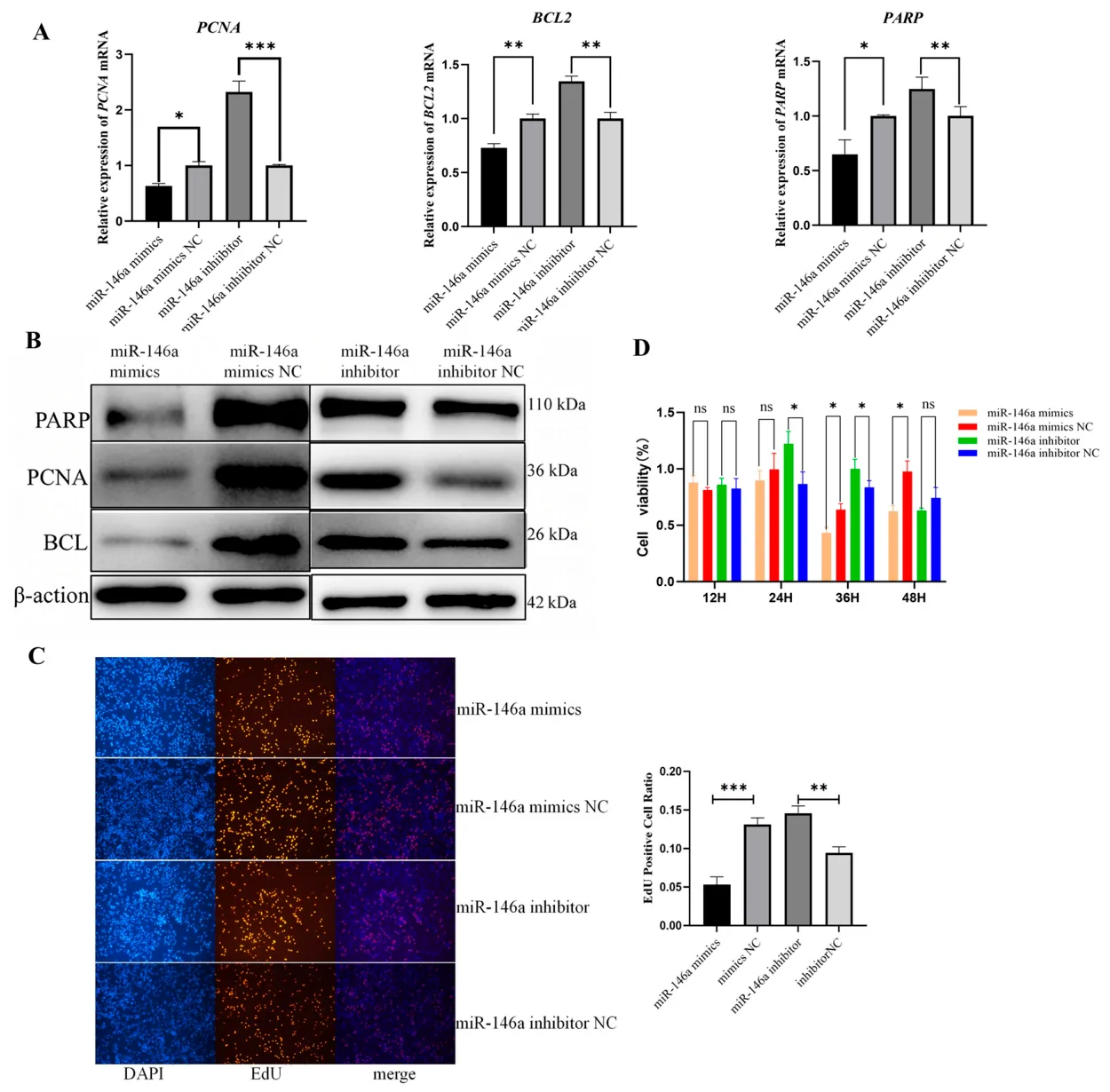
Bta-miR-146a inhibits the proliferation of bovine immature SCs. (**A**) RT-qPCR analysis of proliferation-related genes *PCNA*, *BCL2*, and *PARP*. (**B**) Western blot analysis and quantitative protein expression of PCNA, BCL2, and PARP, normalized to β-actin. (**C**) EdU staining assay (red/orange: proliferating cells; blue: DAPI). Images were captured at 4× magnification; scale bar = 100 μm. (**D**) CCK-8 cell viability assay at different time points. Values are mean ± SEM (n = 3). *, *p* < 0.05; **, *p* < 0.01; ***, *p* < 0.001 versus respective NC.

To explore the regulatory role of Bta-miR-146a in SCs proliferation, Bta-miR-146a mimics or inhibitors were transfected into bovine immature SCs to modulate Bta-miR-146a levels. Cell proliferation was assessed using RT-qPCR, Western blotting, EdU staining, and CCK-8 assays. RT-qPCR analysis (Figure 2A) revealed that transfection with Bta-miR-146a mimics significantly downregulated the mRNA expression of the proliferation marker genes PCNA and BCL2 (*p* < 0.05), whereas inhibition of Bta-miR-146a levels significantly upregulated the mRNA expression of *PCNA*, *PARP*, and *BCL2* (*p* < 0.05). Western blot analysis (Figure 2B) confirmed that overexpression of Bta-miR-146a significantly reduced the protein levels of PCNA, PARP, and BCL2 (*p* < 0.05), whereas inhibition of Bta-miR-146a levels significantly increased their levels (*p* < 0.05). EdU staining further validated these findings, revealing a significant reduction in the proliferation rate in cells transfected with Bta-miR-146a mimics compared with that in the mimic NC group (*p* < 0.01; Figure 2C), whereas inhibition of Bta-miR-146a levels significantly increased proliferation compared with that in the inhibitor NC group (*p* < 0.05; Figure 2C). Additionally, CCK-8 assays conducted 36 h post-transfection demonstrated that Bta-miR-146a overexpression significantly reduced cell viability (*p* < 0.05; Figure 2D), whereas inhibition of Bta-miR-146a levels significantly increased cell viability (*p* < 0.05; Figure 2D). Collectively, these results indicate that Bta-miR-146a overexpression suppresses the proliferation of bovine immature SCs, whereas its inhibition promotes proliferation.

### 2.3. Bta-miR-146a Promotes Apoptosis in Immature Bovine SCs

**Figure 3:**
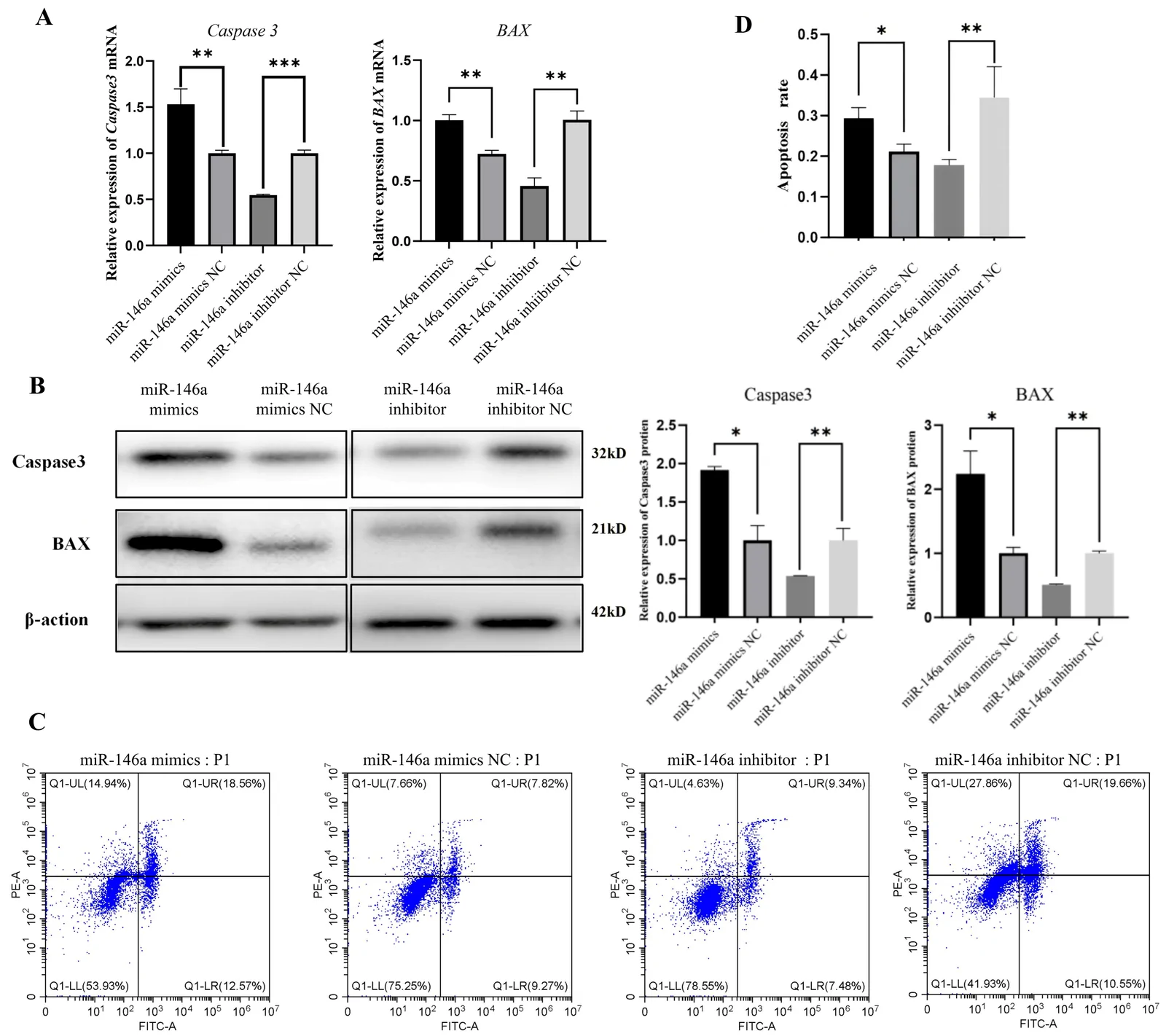
Bta-miR-146a promotes apoptosis in bovine immature SCs. (**A**) RT-qPCR analysis of apoptosis-related genes *Caspase 3* and *BAX*. (**B**) Western blot analysis and quantitative protein expression of Caspase 3 and BAX, normalized to β-actin. (**C**) Flow cytometry analysis using Annexin V-FITC/PI staining. (**D**) Quantification of total apoptotic rate. Values are mean ± SEM (n = 3). *, *p* < 0.05; **, *p* < 0.01; ***, *p* < 0.001 versus respective NC.

To investigate whether Bta-miR-146a regulates apoptosis in bovine immature SCs, cells were transfected with Bta-miR-146a mimics to overexpress Bta-miR-146a. RT-qPCR analysis (Figure 3A) revealed that Bta-miR-146a overexpression significantly increased the mRNA expression of the apoptosis marker genes *Caspase 3* and *BAX* (*p* < 0.05), whereas inhibition of Bta-miR-146a levels significantly reduced their mRNA expression (*p* < 0.05). Western blot analysis (Figure 3B) revealed that Bta-miR-146a overexpression significantly increased the protein levels of Caspase 3 and BAX compared with those in the control group (*p* < 0.05), whereas inhibition of Bta-miR-146a expression significantly decreased their protein expression (*p* < 0.05). To further confirm the effect of Bta-miR-146a on apoptosis, cells were stained with FITC/PI and analyzed by flow cytometry. As shown in Figure 3C, compared with the control treatment, Bta-miR-146a overexpression significantly increased the apoptosis rate (*p* < 0.05), whereas inhibition of Bta-miR-146a levels significantly reduced the apoptosis rate (*p* < 0.05). These findings demonstrate that Bta-miR-146a overexpression promotes apoptosis in immature bovine SCs by upregulating the expression of apoptosis-related genes, while its inhibition suppresses apoptosis.

### 2.4. Effects of Bta-miR-146a on Secretory Factors in Bovine Immature SCs

To evaluate the effect of Bta-miR-146a on the expression of secretory factors in bovine immature SCs, RT-qPCR was used to analyze the expression of *GDNF*, *CXCL12*, *FGF2*, and *BMP4*. As shown in Figure S1A, overexpression of Bta-miR-146a significantly reduced the mRNA expression of *GDNF*, *CXCL12* (*p* < 0.01) and *BMP4* (*p* < 0.05), whereas the mRNA expression of *FGF2* slightly but nonsignificantly decreased (*p* > 0.05). Conversely, compared with the control treatment, the inhibition of Bta-miR-146a levels significantly increased the mRNA expression of *GDNF*, *CXCL12*, and *FGF2* (*p* < 0.05). Western blot analysis (Figure S1B) demonstrated that Bta-miR-146a overexpression significantly reduced the expression of androgen-binding protein ABP (*p* < 0.05), whereas inhibition of Bta-miR-146a expression significantly increased ABP levels (*p* < 0.05). These results indicate that Bta-miR-146a suppresses the expression of specific secretory factors in bovine immature SCs, potentially affecting their role in supporting spermatogenesis.

### 2.5. SMAD4 Is a Direct Target of Bta-miR-146a

**Figure 4:**
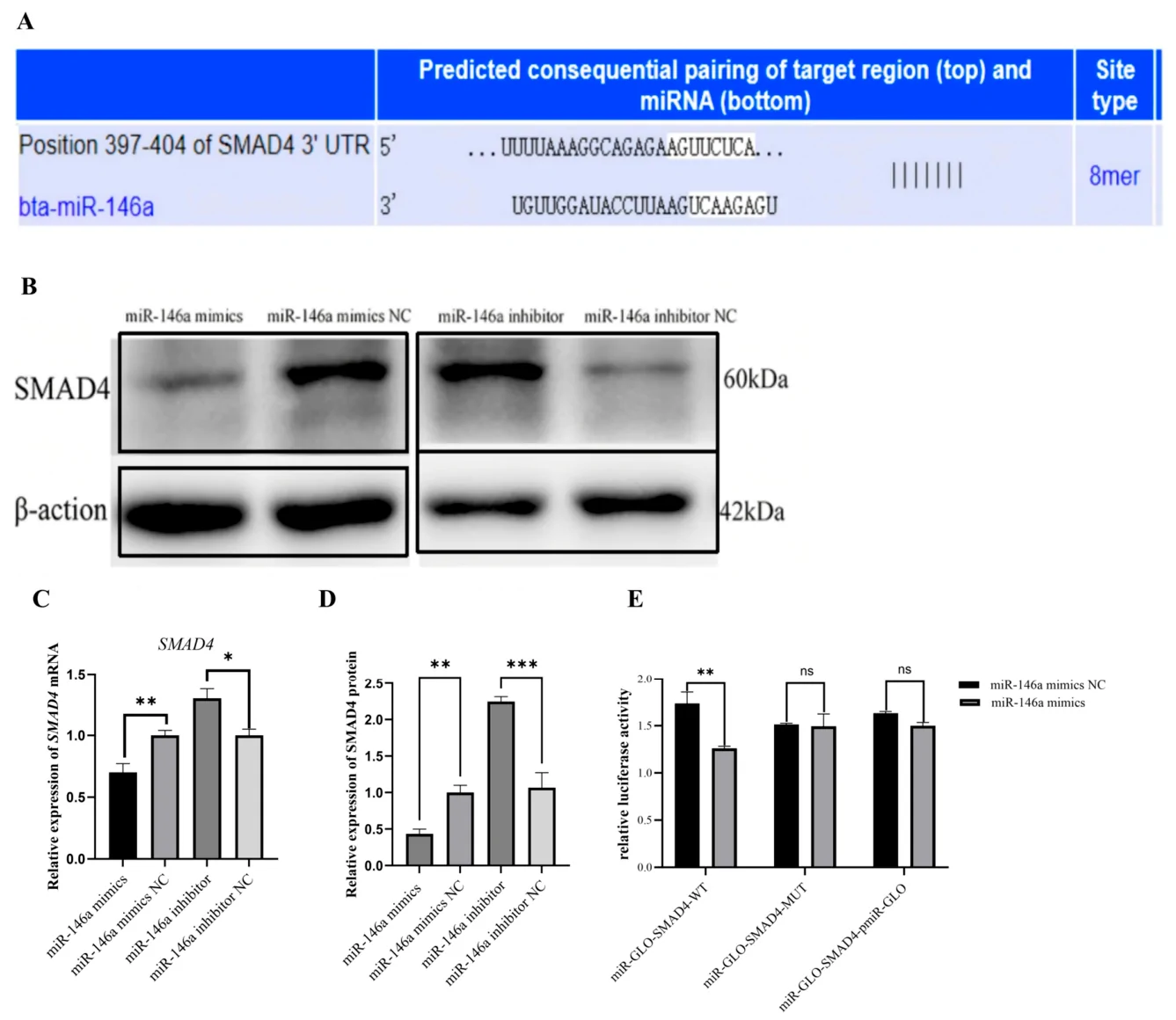
SMAD4 is a direct target of Bta-miR-146a in bovine immature SCs. (**A**) Predicted binding site of Bta-miR-146a in the 3′UTR of *SMAD4*. (**B**) Western blot analysis of SMAD4 protein levels. (**C**) Relative mRNA expression of *SMAD4* detected by RT-qPCR. (**D**) Quantification of SMAD4 protein levels. (**E**) Dual-luciferase reporter assay. Values are mean ± SEM (n = 3). *, *p* < 0.05; **, *p* < 0.01; ***, *p* < 0.001 versus respective NC.

TargetScan analysis identified SMAD4 as a potential target of Bta-miR-146a, with a predicted binding site in its 3′UTR (Figure 4A). Transfection of Bta-miR-146a mimics into bovine immature testicular Sertoli cells significantly reduced both *SMAD4* mRNA (*p* < 0.01) and protein levels (*p* < 0.01), whereas transfection of Bta-miR-146a inhibitors increased *SMAD4* mRNA (*p* < 0.05) and protein expression (*p* < 0.01) (Figure 4B–D). To further confirm the direct interaction between Bta-miR-146a and the SMAD4 3′UTR, dual-luciferase reporter assays were performed. Co-transfection of Bta-miR-146a mimics with pmirGLO-SMAD4-WT significantly decreased luciferase activity compared with the negative control (*p* < 0.01). In contrast, co-transfection with pmirGLO-SMAD4-MUT or the empty pmirGLO vector did not significantly affect luciferase activity (Figure 4E). These results demonstrate that Bta-miR-146a specifically binds to the 3′UTR of SMAD4 and suppresses its expression.

### 2.6. Effects of SMAD4 Interference and Overexpression on the Proliferation of Bovine Immature SCs

**Figure 5:**
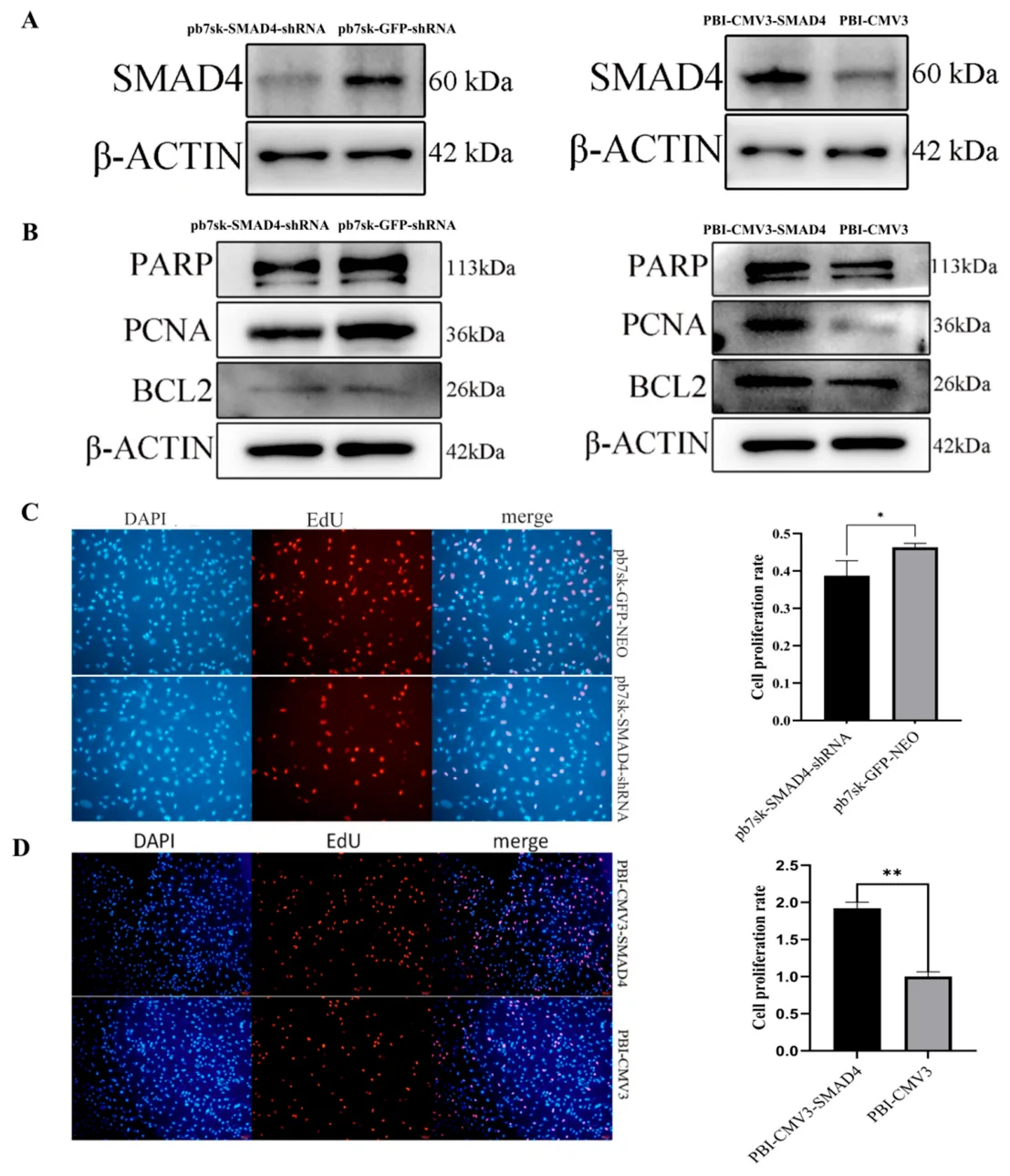
Effects of SMAD4 interference and overexpression on the proliferation of bovine immature SCs. (**A**) Western blot analysis of SMAD4 protein levels after transfection with pb7sk-GFP-SMAD4-shRNA (**left**) or PBI-CMV3-SMAD4 (**right**), with corresponding controls (pb7sk-GFP-NEO and PBI-CMV3). β-actin was used as the loading control. (**B**) Western blot analysis of the proliferation-related proteins PARP, PCNA, and BCL2 under SMAD4 interference (**left**) and overexpression (**right**) conditions. β-actin served as the loading control. (**C**) Representative images of EdU staining (red) and DAPI nuclear staining (blue) in SMAD4-interfered cells and quantification of the cell proliferation rate. Images were captured at 20× magnification; scale bar = 100 μm. (**D**) Representative images of EdU staining and quantification of the cell proliferation rate in SMAD4-overexpressing cells. Images were captured at 4× magnification; scale bar = 100 μm. Values are presented as mean ± SEM (n = 3). *, *p* < 0.05; **, *p* < 0.01 versus the respective control. Quantitative analysis of the Western blot bands and corresponding mRNA expression levels of *SMAD4*, *PARP*, *PCNA*, and *BCL2* are shown in Supplementary Figure S2.

To evaluate the role of SMAD4 in regulating the proliferation of bovine immature SCs, cells were transfected with either the pb7sk-GFP-SMAD4-shRNA vector (SMAD4 interference) or the PBI-CMV3-SMAD4 vector (SMAD4 overexpression), along with their respective controls (pb7sk-GFP-NEO and PBI-CMV3). Western blot analysis confirmed successful modulation of SMAD4 protein levels: SMAD4 interference significantly reduced SMAD4 expression, whereas SMAD4 overexpression markedly increased it (Figure 5A).

RT-qPCR analysis revealed that SMAD4 interference reduced *SMAD*4 mRNA levels by more than 60% (*p* < 0.05) and significantly downregulated the mRNA expression of the proliferation-related genes *PARP*, *PCNA*, and *BCL2* (*p* < 0.05). In contrast, SMAD4 overexpression significantly increased SMAD4 mRNA levels (*p* < 0.001) and upregulated the mRNA expression of *PARP*, *PCNA*, and *BCL2* (*p* < 0.05). Corresponding quantitative analysis of the Western blot bands for SMAD4, PARP, PCNA, and BCL2 is shown in Supplementary Figure S2.

Consistently, Western blot results showed that SMAD4 interference decreased the protein levels of PARP, PCNA, and BCL2, while SMAD4 overexpression elevated the expression of these proteins (Figure 5B).

EdU incorporation assays further demonstrated that SMAD4 interference significantly reduced the cell proliferation rate compared with the control group (*p* < 0.05; Figure 5C), whereas SMAD4 overexpression significantly enhanced the proliferation rate (*p* < 0.01; Figure 5D). Collectively, these results indicate that SMAD4 positively regulates the proliferation of bovine immature Sertoli cells.

### 2.7. Effects of SMAD4 Interference and Overexpression on Apoptosis and Secretory Function of Bovine Immature SCs

To further investigate the role of SMAD4 in regulating apoptosis and secretory function in bovine immature SCs, the same transfection groups (pb7sk-GFP-SMAD4-shRNA vs. pb7sk-GFP-NEO; PBI-CMV3-SMAD4 vs. PBI-CMV3) were analyzed.

**Figure 6:**
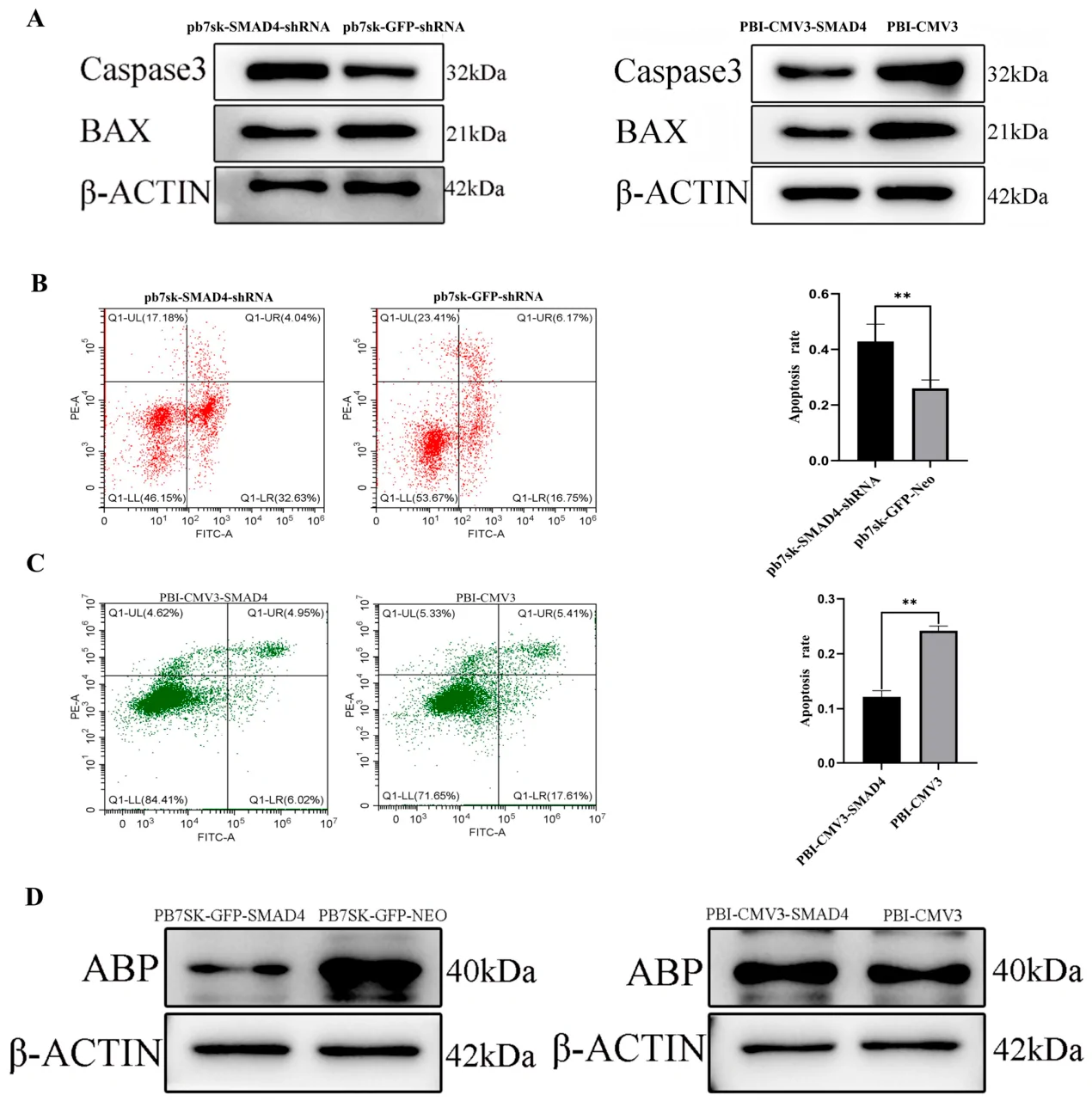
Effects of SMAD4 interference and overexpression on apoptosis and secretory function of bovine immature SCs. (**A**) Western blot analysis of the pro-apoptotic proteins Caspase3 and BAX after SMAD4 interference (**left**: pb7sk-SMAD4-shRNA vs. pb7sk-GFP-shRNA) and overexpression (**right**: PBI-CMV3-SMAD4 vs. PBI-CMV3). β-actin was used as the loading control. (**B**) Representative flow cytometry plots of Annexin V-FITC/PI staining and quantification of the apoptosis rate in SMAD4-interfered cells. (**C**) Representative flow cytometry plots and quantification of the apoptosis rate in SMAD4-overexpressing cells. (**D**) Western blot analysis of the secretory protein ABP under SMAD4 interference (**left**) and overexpression (**right**) conditions. β-actin served as the loading control. Values are mean ± SEM (n = 3). **, *p* < 0.01.

Western blot analysis showed that SMAD4 interference increased the protein levels of the pro-apoptotic markers Caspase3 and BAX, whereas SMAD4 overexpression decreased their protein levels (Figure 6A). RT-qPCR analysis revealed that SMAD4 interference significantly upregulated *Caspase3* mRNA expression (*p* < 0.05), while the increase in *BAX* mRNA was not statistically significant. In contrast, SMAD4 overexpression significantly reduced the mRNA levels of both *Caspase3* and *BAX* (*p* < 0.05). Quantitative analysis of the Western blot bands and the corresponding mRNA expression data are presented in Supplementary Figure S3.

Flow cytometry analysis using Annexin V-FITC/PI staining further confirmed these findings. SMAD4 interference significantly increased the apoptosis rate compared with the control group (*p* < 0.01; Figure 6B), whereas SMAD4 overexpression significantly decreased the apoptosis rate (*p* < 0.01; Figure 6C).

In addition, SMAD4 interference reduced the protein expression of the Sertoli cell secretory factor ABP, while SMAD4 overexpression increased ABP protein levels (Figure 6D). Corresponding quantitative data are shown in Supplementary Figure S3.

Collectively, these results demonstrate that SMAD4 inhibits apoptosis and promotes the secretory function of bovine immature Sertoli cells.

Values are presented as mean ± SEM (n = 3). **, *p* < 0.01 versus the respective control. Quantitative analysis of the Western blot bands and corresponding mRNA expression levels of *Caspase3* and *BAX* are shown in Supplementary Figure S3.

### 2.8. Regulation of the TGFβ/MAPK Signaling Pathway by SMAD4

Previous studies have confirmed the regulatory role of SMAD4 in SCs proliferation and apoptosis, with KEGG analysis indicating its influence on the MAPK pathway via TGFβ. To further verify this relationship in bovine immature SCs, the expression of key TGF-β/MAPK pathway components was examined after SMAD4 interference and overexpression.

**Figure 7:**
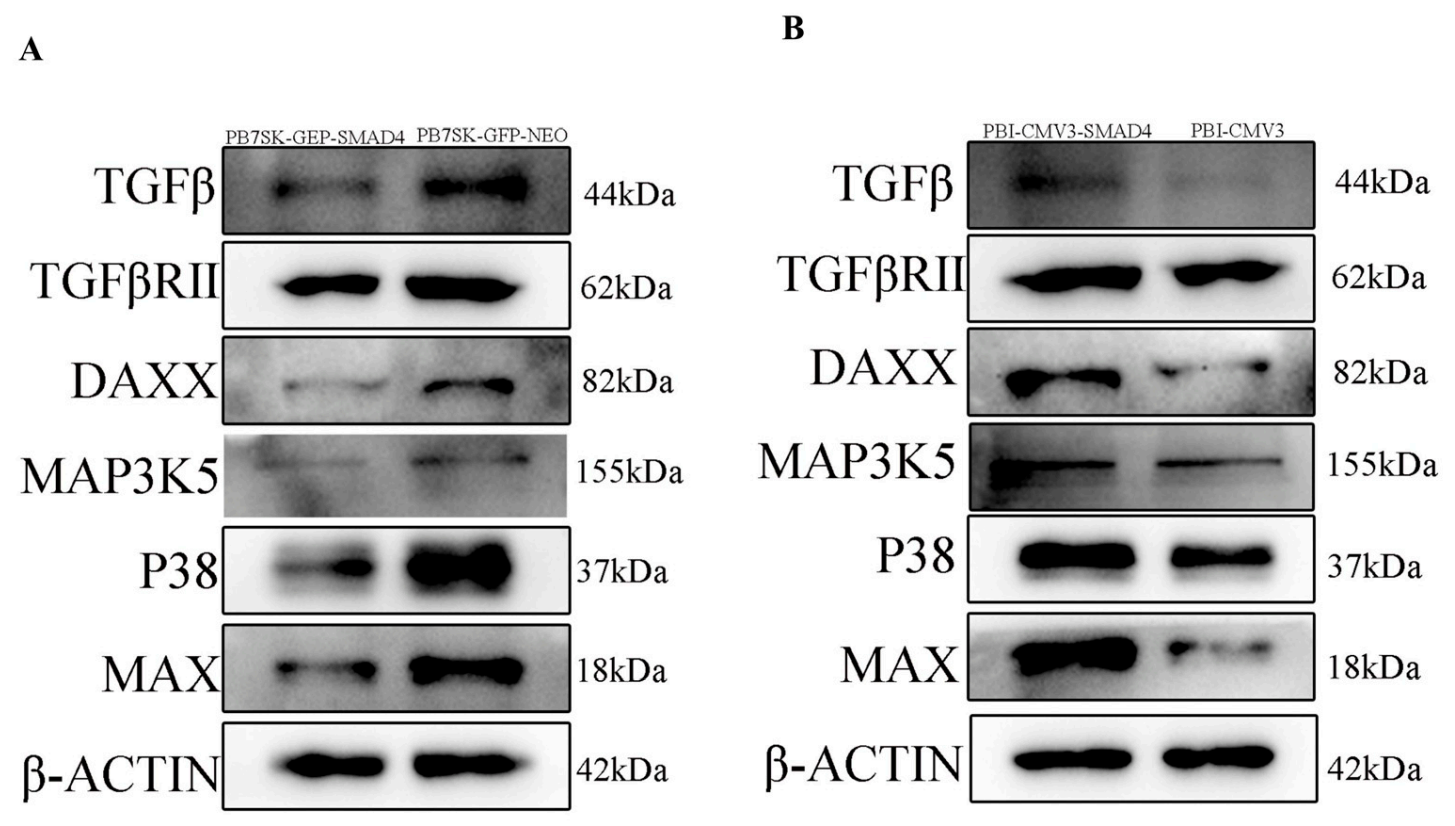
SMAD4 regulates the TGF-β/MAPK signaling pathway in bovine immature SCs. (**A**) Western blot analysis of key TGF-β/MAPK pathway proteins (TGFβ, TGFβRII, DAXX, MAP3K5, P38, and MAX) after SMAD4 interference (PB7SK-GFP-SMAD4 vs. PB7SK-GFP-NEO). β-actin was used as the loading control. (**B**) Western blot analysis of the same proteins after SMAD4 overexpression (PBI-CMV3-SMAD4 vs. PBI-CMV3). β-actin served as the loading control.

RT-qPCR analysis following SMAD4 interference revealed significant downregulation of the mRNA expression of *TGFβ*, *DAXX*, *MAP3K5*, *P38*, *DDIT3*, and *MAX* (*p* < 0.05). Western blot analysis confirmed that the protein levels of TGFβ, TGFβRII, MAP3K5, and MAX were reduced (*p* < 0.05), whereas those of DAXX and P38 were highly significantly decreased (*p* < 0.01) (Figure 7A).

Conversely, transfection with PBI-CMV3-SMAD4 significantly increased the mRNA expression of *TGFβ*, *DAXX*, *MAP3K5*, *P38*, and *MAX* (*p* < 0.05) and elevated the protein levels of TGFβ, TGFβRII, DAXX, MAP3K5, P38, and MAX (*p* < 0.05) compared with the PBI-CMV3 control group (Figure 7B). Quantitative analysis of the Western blot bands and the corresponding mRNA expression data are shown in Supplementary Figure S4. These findings demonstrate that SMAD4 positively regulates the TGF-β/MAPK signaling pathway in bovine immature SCs, with its suppression decreasing and overexpression increasing pathway activity.

## 3. Discussion

Dysregulation of SCs’ proliferation and apoptosis can lead to germ cell loss and male infertility, highlighting the need to elucidate the molecular mechanisms governing these processes [28]. MicroRNAs are crucial posttranscriptional regulators, and as such have been shown to modulate SCs’ function by targeting genes involved in proliferation and apoptosis [29,30]. In this study, Bta-miR-146a, which is differentially expressed in immature and mature bovine testicular tissues, was investigated for its regulatory role in bovine testicular immature SCs. Our findings demonstrate that Bta-miR-146a suppresses proliferation and promotes apoptosis by targeting SMAD4, a key mediator of the TGFβ/MAPK signaling pathway, providing novel insights into the molecular mechanisms by which it affects bovine testicular development.

Overexpression of Bta-miR-146a significantly reduced the expression of proliferation markers (PCNA, PARP, and BCL2) and SCs-specific secretory factors (GDNF, CXCL12, FGF2, and BMP4) at the mRNA level, as well as ABP protein expression, while increasing the expression of apoptosis markers (Caspase3 and BAX) and the apoptosis rate, as confirmed by EdU staining, CCK-8 assays, RT-qPCR, Western blotting, and flow cytometry (Figure 2, Figure 3 and Figure S1). Conversely, inhibition of Bta-miR-146a expression increased proliferation and reduced apoptosis, indicating that Bta-miR-146a plays a negative regulatory role in SCs’ growth and survival. These results align with those of previous studies in bovine immature SCs, in which miRNAs such as miR-196a and miR-362 were shown to regulate proliferation and apoptosis by targeting specific genes [31,32].

Target prediction using TargetScan7.2 and validation via dual-luciferase reporter assays confirmed that SMAD4 is a direct target of Bta-miR-146a, with specific binding to its 3′UTR. Overexpression of Bta-miR-146a significantly decreased SMAD4 mRNA and protein levels, whereas inhibition had the opposite effect, which is consistent with findings in human dermal fibroblasts [33]. SMAD4, a central transcription factor in the TGF-β signaling pathway, forms complexes with SMAD2/3 to regulate the expression of genes critical for cell proliferation, apoptosis, and differentiation [34]. Its role in SCs extends to maintaining blood–testis barrier (BTB) integrity and supporting spermatogenesis, as evidenced by studies showing impaired BTB and spermatogenic defects in SMAD4-knockout mice [26,35]. Our experiments further demonstrated that SMAD4 interference inhibited proliferation and promoted apoptosis by downregulating the expression of *PCNA*, *PARP*, *BCL*2, and *CyclinD1* and upregulating the expression of *Caspase3* and *BAX*, with opposite effects observed upon SMAD4 overexpression. Consistent with our findings, recent studies have shown that other miRNAs, such as miR-181c/d, also repress Sertoli cell proliferation and promote apoptosis by targeting specific downstream genes, highlighting the conserved role of miRNAs in regulating Sertoli cell fate during mammalian spermatogenesis [36]. These findings confirm the role of SMAD4 as a positive regulator of SCs’ proliferation and survival, likely through its modulation of TGF-β signaling.

TGF-β/MAPK signaling has emerged as a critical mediator of the effects of SMAD4 on bovine SCs. KEGG pathway analysis further revealed that SMAD4 interacts with the MAPK cascade via TGF-β signaling, a mechanism that has been largely underexplored in bovine species to date. RT-qPCR and Western blot analyses revealed that SMAD4 interference significantly reduced the mRNA and protein levels of TGFβ/MAPK pathway-related genes (*TGFβ*, *TGF-βRII*, *DAXX*, *MAP3K5*, *P38*, *DDIT3*, and *MAX*), with the decreases in the expression of *DAXX* and *P38* being particularly pronounced (*p* < 0.01) (Figure 7). In contrast, SMAD4 overexpression significantly upregulated the expression of these genes (*p* < 0.05) (Figure 7B). These results suggest that SMAD4 enhances TGFβ/MAPK signaling, likely through direct transcriptional regulation of genes such as ASK1 (MAP3K5) and TAK1, as supported by prior ChIP analyses showing SMAD4 enrichment at the *ASK1* promoter [37,38]. Additionally, SMAD4 may indirectly influence MAPK activity via TRAF6 or CBP/p300, which is consistent with its role in other cell types [24,39]. The p38 MAPK pathway has been recognized as a critical regulator of testis development and Sertoli cell function, influencing both the proliferation of immature Sertoli cells and the maintenance of their mature functions [40]. Our observation that SMAD4 modulates the expression of key components of this pathway, including P38, further supports the importance of the TGF-β/MAPK axis in bovine Sertoli cells. The interplay between the TGF-β and p38 MAPK pathways, mediated by SMAD4, likely contributes to the observed effects on SCs’ proliferation and apoptosis, which aligns with reports of the role of SMAD4 in p38 MAPK activation in hepatocytes and osteoblasts [41,42].

The regulation of SCs-specific secretory factors (*CXCL12*, *BMP4*, and *ABP*) by SMAD4 further highlights its role in testicular function. SMAD4 interference decreased the expression of these factors, while SMAD4 overexpression increased their expression (Figure 6 and Figure 7), suggesting that SMAD4 supports the secretory capacity of SCs, which is critical for nurturing germ cells during spermatogenesis [43]. These findings are consistent with those of studies showing that SMAD4-mediated BMP signaling promotes spermatogonial stem cell self-renewal and differentiation [44].

Despite these findings, the precise molecular mechanisms by which Bta-miR-146a and SMAD4 regulate bovine SCs’ function remain unclear. The observed effects may involve additional pathways, such as Wnt/β-catenin or Notch, which can interact with TGF-β/MAPK signaling [45]. Species-specific differences and the unique microenvironment of bovine SCs warrant further investigation, potentially through single-cell sequencing or proteomics, to elucidate additional targets and interactions. Moreover, while our study established the role of Bta-miR-146a in bovine testicular development, its therapeutic potential in addressing male infertility and optimizing reproductive outcomes in livestock requires further exploration.

It should be noted that the present study lacks a formal rescue experiment (e.g., overexpression of bta-miR-146a in the SMAD4-knockdown background) to definitively establish that the effects of bta-miR-146a are mediated solely through SMAD4. In addition, although we observed changes in the expression levels of several TGF-β/MAPK pathway components, the phosphorylation status of key MAPK proteins (such as p38) was not examined, which would more directly reflect pathway activation. These limitations will be addressed in future studies.

In conclusion, Bta-miR-146a inhibits proliferation and promotes apoptosis in bovine testicular immature SCs by targeting SMAD4, which positively regulates the TGFβ/MAPK signaling pathway and SCs’ secretory functions. These findings provide a foundation for understanding the molecular regulation of testicular development in cattle and highlight the potential of miRNA-based interventions in reproductive biology.

## 4. Materials and Methods

### 4.1. Cell Culture

Immature SCs (SCs) were isolated from the testes of newborn (1–3 days old) healthy Chinese Holstein cattle. All animal procedures were approved by the Animal Ethics Committee of Guangdong Ocean University (Approval No. 20240301) and performed in strict accordance with the Guidelines for the Care and Use of Laboratory Animals of Guangdong Ocean University and the national regulations of China.

Cells were cultured in DMEM/F-12 medium (BasalMedia, Shanghai, China, Cat. No. L310KJ) supplemented with 10% fetal bovine serum (ZETA LIFE, Atlanta, GA, USA, Cat. No. Z7010FBS or Z7181FBS-500), 100 U/mL penicillin, and 100 μg/mL streptomycin at 37 °C in a humidified atmosphere containing 5.0% CO_2_. The medium was changed every 2 days. When cells reached 70–80% confluence, they were passaged using 0.25% trypsin-EDTA solution (Sangon Biotech, Shanghai, China, Cat. No. E607002). All experiments were performed using cells between passages 3 and 6 to maintain their immature characteristics.

### 4.2. Transfection of miR-146a-5p Mimics/Inhibitors and Their NC

Bta-miR-146a mimics (chemically synthesized double-stranded RNA oligonucleotides that mimic endogenous mature Bta-miR-146a) and inhibitors (chemically synthesized single-stranded antisense oligonucleotides that specifically bind and sequester endogenous Bta-miR-146a), together with their respective negative controls (mimic NC and inhibitor NC), were designed and synthesized by GenePharma Co., Ltd. (Suzhou, China). For SMAD4 knockdown, shRNA sequences targeting bovine SMAD4 (NM_174047.2) were designed using the Thermo Fisher RNAi Designer tool (Thermo Fisher Scientific, Waltham, MA, USA) and cloned into the pGPU6/GFP/Neo vector (GenePharma, Shanghai, China). For SMAD4 overexpression, the full-length coding sequence (CDS) of bovine SMAD4 was amplified by PCR and inserted into the PBI-CMV3 expression vector (Clontech/Takara, Mountain View, CA, USA, Cat. No. 631630 or 631632).

Transfection was performed when cells reached 60–70% confluence in 6-well plates (for RNA/protein extraction and functional assays) or 96-well plates (for CCK-8). Lipofectamine 3000 (Thermo Fisher Scientific, Waltham, MA, USA, Cat. No. L3000015) was used according to the manufacturer’s instructions. Briefly, 2.5 μg plasmid DNA or 50–100 nM mimics/inhibitors and 5.0 μL P3000 reagent were diluted in 125 μL Opti-MEM (or serum-free DMEM/F-12), mixed with 3.75–7.5 μL Lipofectamine 3000 diluted in 125 μL serum-free medium, and incubated for 15 min at room temperature. The complexes were added to cells and incubated for 4–6 h, after which the medium was replaced with complete growth medium. Transfection efficiency was verified by fluorescence microscopy (for GFP-containing vectors) and RT-qPCR 36 h post-transfection. All transfections were performed in triplicate and repeated at least three independent times.

### 4.3. Total RNA Extraction, RNA Reverse Transcription and RT-qPCR

Total RNA was extracted from transfected SCs 36 h post-transfection using AG RNAex Pro reagent (Accurate Biology, Changsha, Hunan, China, Cat. No. AG21102) following the manufacturer’s protocol. RNA concentration and purity were assessed using a NanoDrop Lite spectrophotometer (Thermo Fisher Scientific). Only samples with A260/A280 ratios between 1.8 and 2.0 and clear 28S/18S rRNA bands on 1.0% agarose gel were used for downstream analyses.

For miRNA detection, 1.0 μg total RNA was reverse-transcribed using the miRNA 1st Strand cDNA Synthesis Kit (Vazyme Biotech, Nanjing, China, Cat. No. MR101) with specific stem-loop RT primers for Bta-miR-146a and U6 snRNA (internal control). For mRNA detection, reverse transcription was performed using HiScript III RT SuperMix for RT-qPCR (+gDNA wiper) (Vazyme, Cat. No. R323-01).

Quantitative real-time PCR was performed on a CFX96 Real-Time PCR Detection System (Bio-Rad Laboratories, Hercules, CA, USA) using TB Green^®^ Premix Ex Taq™ II (Tli RNaseH Plus) (TaKaRa, Kusatsu, Japan, Cat. No. RR820A) for mRNA or miRNA Universal SYBR RT-qPCR Master Mix (Vazyme, Cat. No. MQ101) for miRNA. The reaction volume was 20 μL, and the thermal cycling conditions were: 95 °C for 5 min, followed by 40 cycles of 95 °C for 10 s and 60 °C for 30 s, with a final melt curve analysis. All reactions were performed in triplicate. Relative expression levels were calculated using the 2^−ΔΔCt^ method and normalized to U6 or β-actin.

### 4.4. Protein Extraction and Western Blotting

Total protein was extracted 36–48 h post-transfection using RIPA lysis buffer (Meilunbio, Dalian, China, Cat. No. MA0151) supplemented with protease and phosphatase inhibitor cocktails. Protein concentration was determined using the TaKaRa BCA Protein Assay Kit (TaKaRa, Cat. No. T9300A). Equal amounts of protein (20–30 μg per lane) were separated by 10% SDS-PAGE and transferred to PVDF membranes (0.45 μm). Membranes were blocked with 5.0% non-fat milk in TBST for 2 h at room temperature, then incubated overnight at 4.0 °C with primary antibodies against: SMAD4 (Bioss, Beijing, China, bs-23966R), PCNA (Bioss, bs-2006R), BCL2 (Abmart, Shanghai, China, T40056), BAX (Bioss, bs-2880R), Caspase3 (Abmart, T40044), PARP (Abmart, T40050), ABP (Bioss, bs-2410R), TGFβ (Bioss, bs-4538R), TGFβRII (Bioss, bs-2006R), DAXX (Bioss, bs-1975R), MAP3K5 (Wanleibio, Shenyang, China, WL04752), P38 (Wanleibio, WL00764), MAX (Bioss, bs-4122R), and β-actin (Bioss, bs-0061R) as loading control. After washing, membranes were incubated with Multi-rAb HRP-conjugated Goat Anti-Rabbit secondary antibody (Proteintech, Rosemont, IL, USA, RGAR001) for 2 h at room temperature. Signals were visualized using ECL chemiluminescence substrate (Tanon, Shanghai, China, Cat. No. 180-5001) on a Tanon 4600 chemiluminescence imaging system. Band intensities were quantified using ImageJ software 1.54 e (National Institutes of Health, Bethesda, MD, USA).

### 4.5. Cell Proliferation Assays

Cell proliferation was evaluated using two independent methods:

CCK-8 assay: Cells were seeded in 96-well plates at a density of 2000–3000 cells per well. After transfection, 10 μL CCK-8 solution (ZETA LIFE, Cat. No. K009) was added to each well containing 100 μL medium and incubated for 2 h at 37 °C. Absorbance was measured at 450 nm using a BioTek Synergy HTX multi-mode microplate reader (Agilent, Santa Clara, CA, USA) at 12 h intervals.

EdU incorporation assay: Cells seeded in 24-well plates were transfected and cultured for 36 h, then incubated with 50 μM EdU (Cell-Light EdU Apollo567 In Vitro Kit, RiboBio, Guangzhou, China, Cat. No. C10310-1) for 3 h. Cells were fixed with 4% paraformaldehyde, permeabilized with 0.5% Triton X-100, and stained according to the manufacturer’s protocol. Nuclei were counterstained with Hoechst 33342. Images were captured using an Olympus IX71 inverted fluorescence microscope (Olympus Corporation, Tokyo, Japan), and the percentage of EdU-positive cells was calculated from at least five random fields per well.

### 4.6. Cell Apoptosis Assay

Apoptosis was detected using the Annexin V-FITC/PI Apoptosis Detection Kit (Vazyme, Cat. No. A211-01). Briefly, 36 h after transfection, cells were harvested with EDTA-free trypsin, washed twice with cold PBS, and resuspended in 400 μL 1× Binding Buffer. Cells were incubated with 5 μL Annexin V-FITC for 5 min and 10 μL PI for 10 min at room temperature in the dark. Apoptotic cells were immediately analyzed using a CytoFLEX LX flow cytometer (Beckman Coulter, Brea, CA, USA). Data were processed with CytExpert software 2.7. Experiments were repeated three times independently.

### 4.7. Dual-Luciferase Reporter Assay

The wild-type and mutant 3′UTR fragments of bovine SMAD4 containing the predicted Bta-miR-146a binding site were synthesized and cloned into the pmirGLO dual-luciferase reporter vector (Promega, Madison, WI, USA). SCs were co-transfected with 2.5 μg reporter plasmid (pmirGLO-SMAD4-WT or MUT) and 50 nM Bta-miR-146a mimics or NC using Lipofectamine 3000. After 30–36 h, cells were lysed and luciferase activities were measured using the Dual-Luciferase Reporter Assay Kit (Vazyme, Cat. No. DL101 or Promega) on a microplate luminometer. Firefly luciferase activity was normalized to Renilla luciferase activity.

### 4.8. Statistical Analysis

Data were analyzed using one-way ANOVA followed by Student’s *t*-test for two-group comparisons with SPSS 22.0. Results are presented as mean ± SEM from at least three independent experiments. *p* < 0.05 was considered statistically significant.

## 5. Conclusions

In this study, we demonstrated that Bta-miR-146a acts as a key negative regulator of proliferation and a promoter of apoptosis in immature SCs of Chinese Holstein cattle. By directly targeting SMAD4, Bta-miR-146a suppresses the TGF-β/MAPK signaling pathway, leading to downregulation of proliferation-related markers (*PCNA*, *BCL2*, *PARP*) and secretory factors (*GDNF*, *CXCL12*, *BMP4*, *ABP*), while upregulating pro-apoptotic factors (*Caspase 3* and *BAX*). Functional experiments further confirmed that SMAD4 positively regulates SC proliferation and secretory activity and inhibits apoptosis. Collectively, these findings establish the Bta-miR-146a/SMAD4/TGF-β/MAPK axis as an important regulatory network controlling SC fate during early testicular development, providing new molecular insights into bovine spermatogenesis and potential targets for improving male reproductive performance in cattle.

## Data Availability

The original contributions presented in this study are included in the article/Supplementary Materials. Further inquiries can be directed to the corresponding author.

## Supplementary Materials

The following supporting information can be downloaded at: https://www.mdpi.com/article/10.3390/ijms27177554/s1.
