# Supplementary material for: Bta-miR-146a Inhibits Proliferation and Promotes Apoptosis of Bovine Immature Sertoli Cells by Targeting SMAD4 via the TGF-β/MAPK Signaling Pathway
Source: Int J Mol Sci. 2026 Aug 24;27(17):7554. doi: 10.3390/ijms27177554 (PMC13566809; doi:10.3390/ijms27177554)
Supplement: Supplementary file 1 [file ijms-27-07554-s001.zip › Figure S1.pdf]

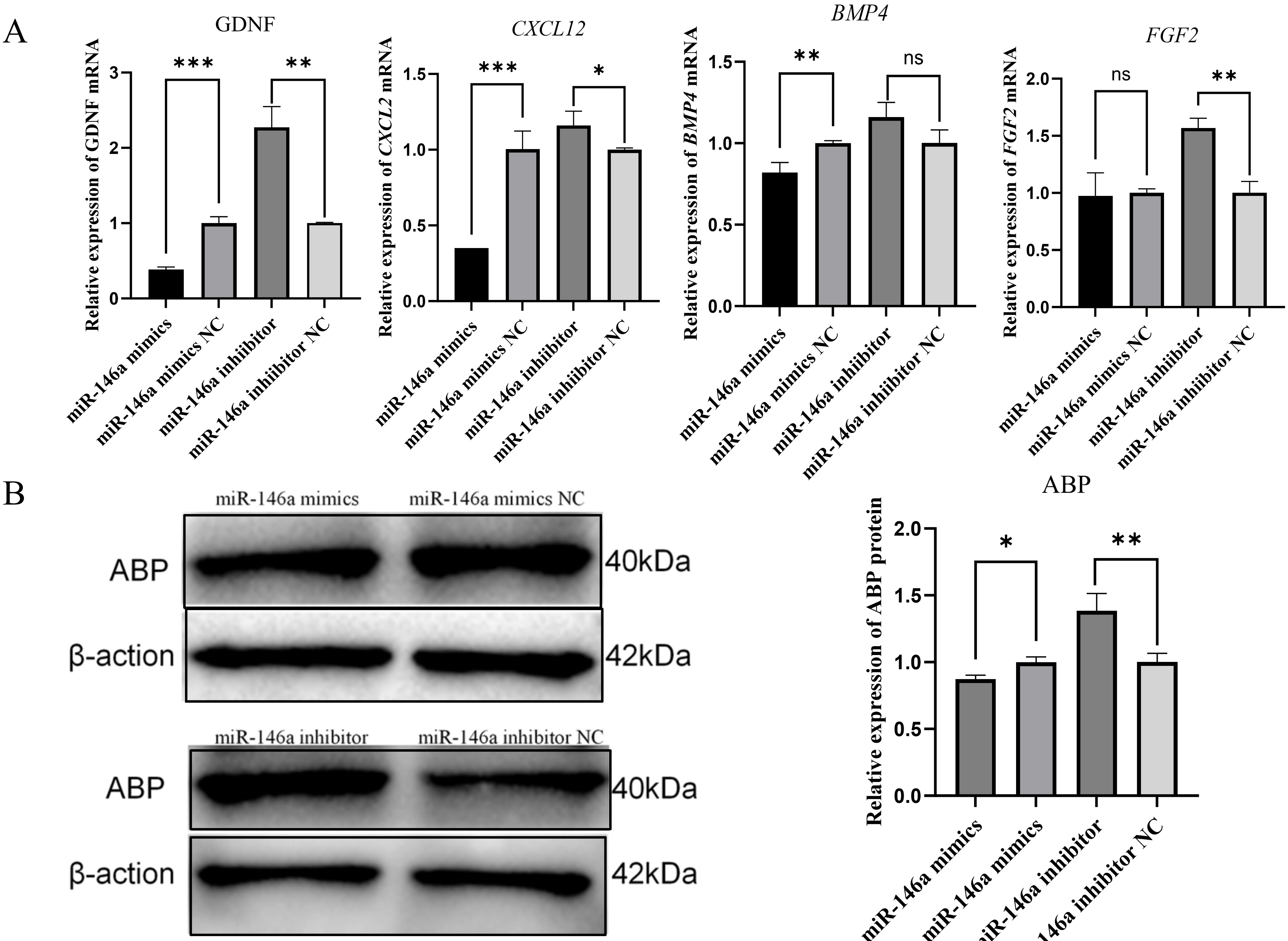

A
GDNF
CXCL12
BMP4
FGF2
Relative expression of GDNF mRNA
Relative expression of CXCL2 mRNA
Relative expression of BMP4 mRNA
Relative expression of FGF2 mRNA
***
**
*
ns
miR-146a mimics
miR-146a mimics NC
miR-146a inhiibitor
miR-146a inhiibitor NC
B
miR-146a mimics
miR-146a mimics NC
miR-146a inhibitor
miR-146a inhibitor NC
ABP
β-action
40kDa
42kDa
ABP
Relative expression of ABP protein
miR-146a mimics
miR-146a mimics
miR-146a inhibitor
miR-146a inhibitor NC
