# Supplementary material for: Bta-miR-146a Inhibits Proliferation and Promotes Apoptosis of Bovine Immature Sertoli Cells by Targeting SMAD4 via the TGF-β/MAPK Signaling Pathway
Source: Int J Mol Sci. 2026 Aug 24;27(17):7554. doi: 10.3390/ijms27177554 (PMC13566809; doi:10.3390/ijms27177554)
Supplement: Supplementary file 1 [file ijms-27-07554-s001.zip › Figure S2.pdf]

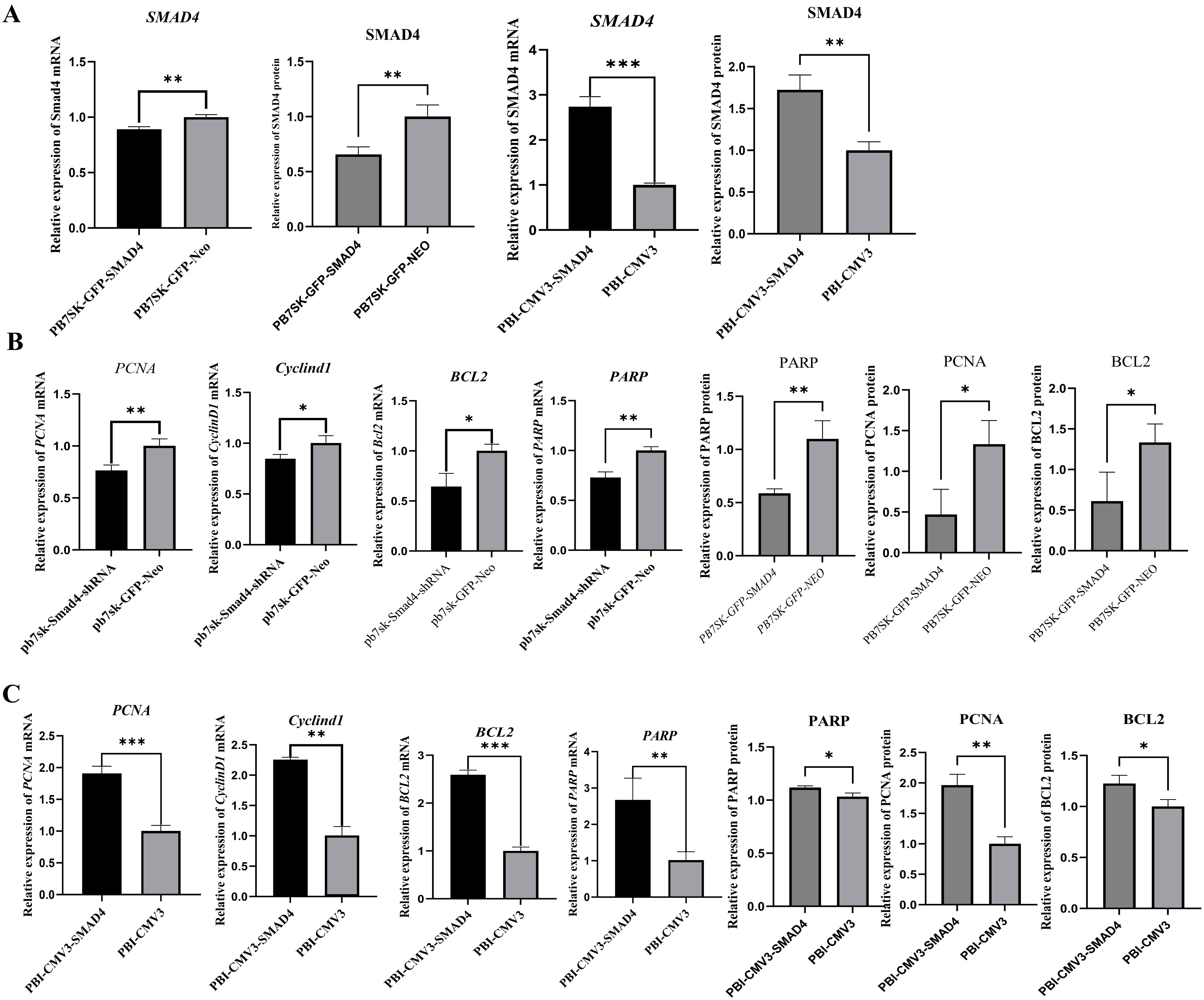
A
SMAD4
Relative expression of Smad4 mRNA
PB7SK-GFP-SMAD4
PB7SK-GFP-Neo
SMAD4
Relative expression of SMAD4 protein
PB7SK-GFP-SMAD4
PB7SK-GFP-NEO
SMAD4
Relative expression of SMAD4 mRNA
PBI-CMV3-SMAD4
PBI-CMV3
SMAD4
Relative expression of SMAD4 protein
PBI-CMV3-SMAD4
PBI-CMV3
B
PCNA
Relative expression of PCNA mRNA
Cyclind1
Relative expression of CyclinD1 mRNA
BCL2
Relative expression of Bcl2 mRNA
PARP
Relative expression of PARP mRNA
pb7sk-Smad4-shRNA
pb7sk-GFP-Neo
PARP
Relative expression of PARP protein
PCNA
Relative expression of PCNA protein
BCL2
Relative expression of BCL2 protein
PB7SK-GFP-SMAD4
PB7SK-GFP-NEO
C
PCNA
Relative expression of PCNA mRNA
Cyclind1
Relative expression of CyclinD1 mRNA
BCL2
Relative expression of BCL2 mRNA
PARP
Relative expression of PARP mRNA
PARP
Relative expression of PARP protein
PCNA
Relative expression of PCNA protein
BCL2
Relative expression of BCL2 protein
PBI-CMV3-SMAD4
PBI-CMV3
