# Supplementary material for: Bta-miR-146a Inhibits Proliferation and Promotes Apoptosis of Bovine Immature Sertoli Cells by Targeting SMAD4 via the TGF-β/MAPK Signaling Pathway
Source: Int J Mol Sci. 2026 Aug 24;27(17):7554. doi: 10.3390/ijms27177554 (PMC13566809; doi:10.3390/ijms27177554)
Supplement: Supplementary file 1 [file ijms-27-07554-s001.zip › Figure S3.pdf]

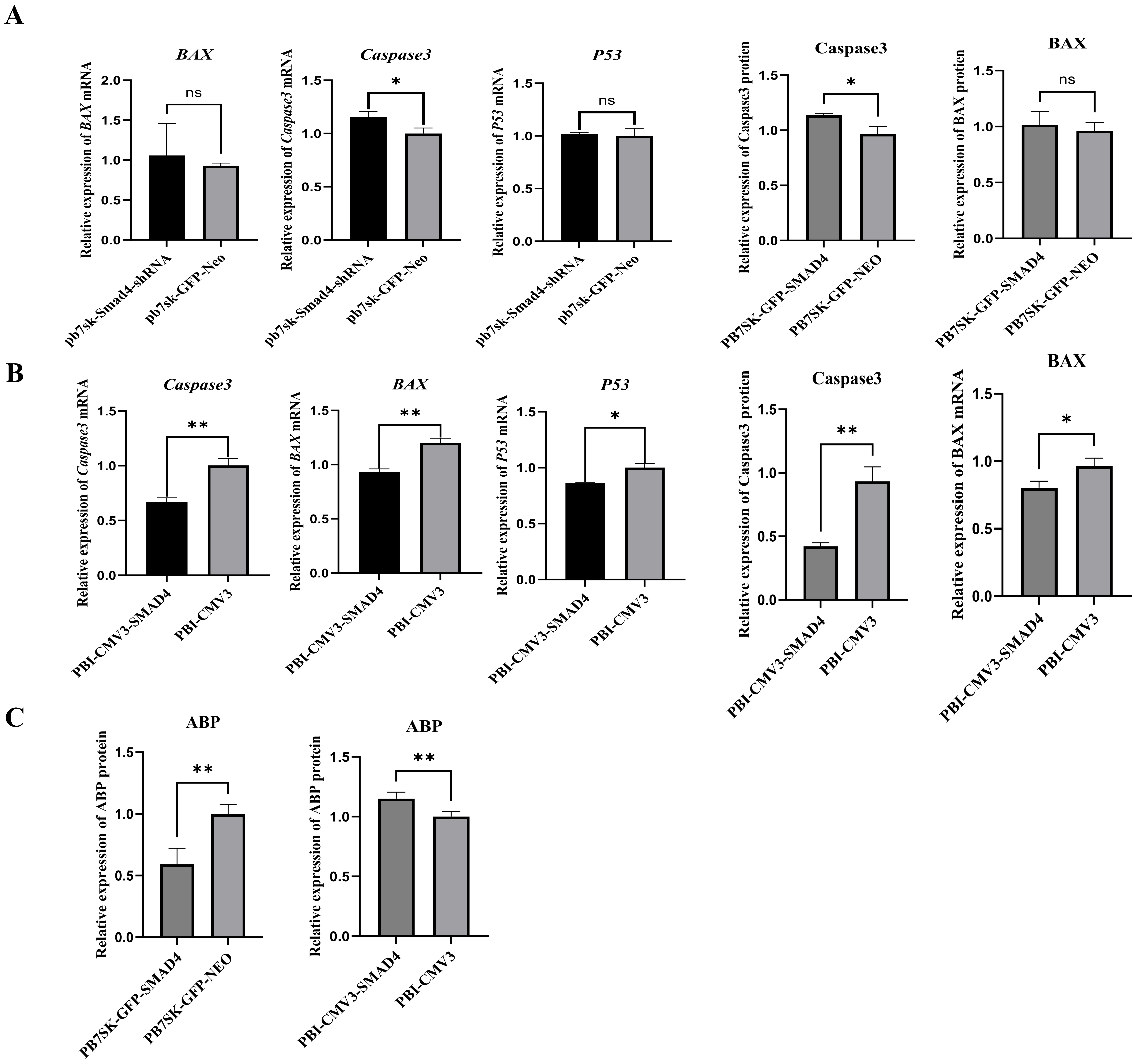
A
BAX
Relative expression of BAX mRNA
ns
pb7sk-Smad4-shRNA
pb7sk-GFP-Neo
Caspase3
Relative expression of Caspase3 mRNA
*
P53
Relative expression of P53 mRNA
ns
Caspase3
Relative expression of Caspase3 protien
*
PB7SK-GFP-SMAD4
PB7SK-GFP-NEO
BAX
Relative expression of BAX protien
ns
B
Caspase3
Relative expression of Caspase3 mRNA
**
PBI-CMV3-SMAD4
PBI-CMV3
BAX
Relative expression of BAX mRNA
**
P53
Relative expression of P53 mRNA
*
Caspase3
Relative expression of Caspase3 protien
**
BAX
Relative expression of BAX mRNA
*
C
ABP
Relative expression of ABP protein
**
PB7SK-GFP-SMAD4
PB7SK-GFP-NEO
ABP
Relative expression of ABP protein
**
PBI-CMV3-SMAD4
PBI-CMV3
