# Supplementary material for: Bta-miR-146a Inhibits Proliferation and Promotes Apoptosis of Bovine Immature Sertoli Cells by Targeting SMAD4 via the TGF-β/MAPK Signaling Pathway
Source: Int J Mol Sci. 2026 Aug 24;27(17):7554. doi: 10.3390/ijms27177554 (PMC13566809; doi:10.3390/ijms27177554)
Supplement: Supplementary file 1 [file ijms-27-07554-s001.zip › Figure S4.pdf]

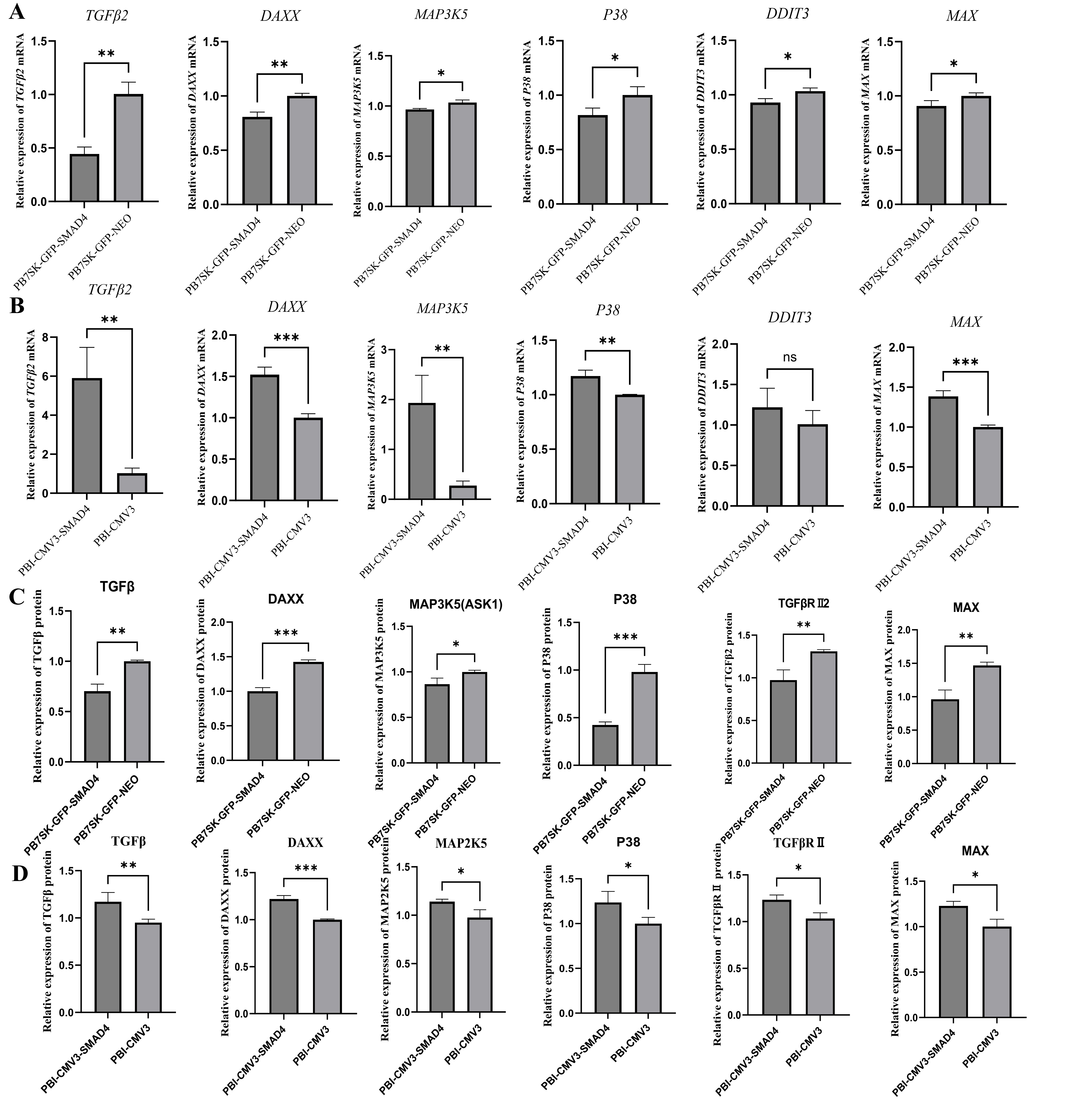

A
TGFβ2
Relative expression of TGFβ2 mRNA
DAXX
Relative expression of DAXX mRNA
MAP3K5
Relative expression of MAP3K5 mRNA
P38
Relative expression of P38 mRNA
DDIT3
Relative expression of DDIT3 mRNA
MAX
Relative expression of MAX mRNA
PB7SK-GFP-SMAD4
PB7SK-GFP-NEO
B
TGFβ2
DAXX
MAP3K5
P38
DDIT3
MAX
ns
PBI-CMV3-SMAD4
PBI-CMV3
C
TGFβ
Relative expression of TGFβ protein
DAXX
Relative expression of DAXX protein
MAP3K5(ASK1)
Relative expression of MAP3K5 protein
P38
Relative expression of P38 protein
TGFβRⅡ2
Relative expression of TGFβ2 protein
MAX
Relative expression of MAX protein
PB7SK-GFP-SMAD4
PB7SK-GFP-NEO
D
TGFβ
Relative expression of TGFβ protein
DAXX
Relative expression of DAXX protein
MAP2K5
Relative expression of MAP2K5 protein
P38
Relative expression of P38 protein
TGFβRⅡ
Relative expression of TGFβRⅡ protein
MAX
Relative expression of MAX protein
PBI-CMV3-SMAD4
PBI-CMV3
