# Supplementary material for: Bta-miR-146a Inhibits Proliferation and Promotes Apoptosis of Bovine Immature Sertoli Cells by Targeting SMAD4 via the TGF-β/MAPK Signaling Pathway
Source: Int J Mol Sci. 2026 Aug 24;27(17):7554. doi: 10.3390/ijms27177554 (PMC13566809; doi:10.3390/ijms27177554)
Supplement: Supplementary file 1 [file ijms-27-07554-s001.zip › Legend.pdf]

Figure S1. Effects of Bta-miR-146a on the expression of secretory factors in bovine immature Sertoli cells.

(A) RT-qPCR analysis of the mRNA expression levels of GDNF, CXCL12, BMP4, and FGF2 after transfection with Bta-miR-146a mimics, mimics NC, inhibitor, or inhibitor NC.

(B) Western blot analysis and quantification of androgen-binding protein (ABP) protein levels (normalized to β-actin) under the same transfection conditions.

Values are presented as mean ± SEM (n = 3). *, p < 0.05; **, p < 0.01; ***, p < 0.001; ns, not significant versus the respective NC.

Figure S2. Quantitative analysis of SMAD4 and proliferation-related gene/protein expression after SMAD4 interference or overexpression in bovine immature Sertoli cells.

(A) Relative mRNA and protein levels of SMAD4 after transfection with pb7sk-GFP-SMAD4-shRNA (interference) versus pb7sk-GFP-NEO, or PBI-CMV3-SMAD4 (overexpression) versus PBI-CMV3.

(B) Relative mRNA levels of PCNA, CyclinD1, BCL2, and PARP, and relative protein levels of PARP, PCNA, and BCL2 after SMAD4 interference.

(C) Relative mRNA levels of PCNA, CyclinD1, BCL2, and PARP, and relative protein levels of PARP, PCNA, and BCL2 after SMAD4 overexpression.

Protein levels were normalized to β-actin. Values are presented as mean ± SEM (n = 3). *, p < 0.05; **, p < 0.01; ***, p < 0.001 versus the respective control.

Figure S3. Quantitative analysis of apoptosis-related gene/protein expression and ABP levels after SMAD4 interference or overexpression in bovine immature Sertoli cells.

(A) Relative mRNA levels of BAX, Caspase3, and P53, and relative protein levels of Caspase3 and BAX after transfection with pb7sk-GFP-SMAD4-shRNA (interference) versus pb7sk-GFP-NEO.

(B) Relative mRNA levels of Caspase3, BAX, and P53, and relative protein levels of Caspase3 and BAX after transfection with PBI-CMV3-SMAD4 (overexpression) versus PBI-CMV3.

(C) Relative protein levels of ABP after SMAD4 interference (left) or overexpression (right).

Protein levels were normalized to β-actin. Values are presented as mean ± SEM (n = 3). *, p < 0.05; **, p < 0.01; ns, not significant versus the respective control.

Figure S4. Quantitative analysis of TGF-β/MAPK signaling pathway components after SMAD4 interference or overexpression in bovine immature Sertoli cells.

(A) Relative mRNA levels of TGFβ2, DAXX, MAP3K5, P38, DDIT3, and MAX after SMAD4 interference (pb7sk-GFP-SMAD4 vs. pb7sk-GFP-NEO).

(B) Relative mRNA levels of the same genes after SMAD4 overexpression (PBI-CMV3-SMAD4 vs. PBI-CMV3).

(C) Relative protein levels of TGFβ, DAXX, MAP3K5 (ASK1), P38, TGFβRII, and MAX after SMAD4 interference.

(D) Relative protein levels of the same proteins after SMAD4 overexpression.

Protein levels were normalized to β-actin. Data are presented as mean ± SEM (n = 3). p < 0.05, *p < 0.01, **p < 0.001; ns, not significant versus the respective control.
